# Applying symptom dynamics to accurately predict influenza virus infection: An international multicenter influenza‐like illness surveillance study

**DOI:** 10.1111/irv.13081

**Published:** 2022-12-08

**Authors:** Jin‐Hua Li, Chin‐Chieh Wu, Yi‐Ju Tseng, Shih‐Tsung Han, Andrew Pekosz, Richard Rothman, Kuan‐Fu Chen

**Affiliations:** ^1^ Clinical Informatics and Medical Statistics Research Center Chang Gung University Taoyuan Taiwan; ^2^ Department of Medical Education Chang Gung Memorial Hospital Chiayi Taiwan; ^3^ Department of Computer Science National Yang Ming Chiao Tung University Hsinchu Taiwan; ^4^ Department of Emergency Medicine Chang Gung Memorial Hospital Linkou Taiwan; ^5^ W. Harry Feinstone Department of Molecular Microbiology and Immunology The Johns Hopkins Bloomberg School of Public Health Baltimore Maryland USA; ^6^ Department of Emergency Medicine Johns Hopkins University School of Medicine Baltimore Maryland USA; ^7^ Department of Emergency Medicine Chang Gung Memorial Hospital Keelung Taiwan

**Keywords:** cough, influenza, influenza‐like illness, symptom prediction, syndromic surveillance

## Abstract

**Background:**

Public health organizations have recommended various definitions of influenza‐like illnesses under the assumption that the symptoms do not change during influenza virus infection. To explore the relationship between symptoms and influenza over time, we analyzed a dataset from an international multicenter prospective emergency department (ED)‐based influenza‐like illness cohort study.

**Methods:**

We recruited patients in the US and Taiwan between 2015 and 2020 with: (1) flu‐like symptoms (fever and cough, headache, or sore throat), (2) absence of any of the respiratory infection symptoms, or (3) positive laboratory test results for influenza from the current ED visit. We evaluated the association between the symptoms and influenza virus infection on different days of illness. The association was evaluated among different subgroups, including different study countries, influenza subtypes, and only patients with influenza.

**Results:**

Among the 2471 recruited patients, 45.7% tested positive for influenza virus. Cough was the most predictive symptom throughout the week (odds ratios [OR]: 7.08–11.15). In general, all symptoms were more predictive during the first 2 days (OR: 1.55–10.28). Upper respiratory symptoms, such as sore throat and productive cough, and general symptoms, such as body ache and fatigue, were more predictive in the first half of the week (OR: 1.51–3.25). Lower respiratory symptoms, such as shortness of breath and wheezing, were more predictive in the second half of the week (OR: 1.52–2.52). Similar trends were observed for most symptoms in the different subgroups.

**Conclusions:**

The time course is an important factor to be considered when evaluating the symptoms of influenza virus infection.

## 1 INTRODUCTION

Influenza viruses infect approximately one billion people annually,^1^ accounting for half of the respiratory infections during the peak of the epidemic.^2^ The World Health Organization (WHO) estimates the annual mortality due to influenza to range from 290,000 to 650,000 deaths globally.^1^ Timely diagnosis of influenza virus infection is vital for preventing severe complications. Despite the wide availability of diagnostic tools for influenza virus, clinical gestalt is still needed to increase the pre‐test probability and help physicians test for specific pathogens.^3^

In the past few decades, many investigators focused on the utility of syndromic surveillance to differentiate influenza from other virus infections.^4^, ^5^, ^6^ Unfortunately, there was no single symptom that could be used to detect influenza virus infection with perfect sensitivity.^7^, ^8^ To improve the accuracy and to standardize cases for investigation, WHO, the Centers for Disease Control and Prevention (CDC), and the European Centre for Disease Prevention and Control (ECDC) utilized and revised different combinations of symptoms to define influenza‐like illness (Table S1). The sensitivities of these definitions, however, are far from perfect, ranging from 32% to 96% (Table S2).

Some researchers have attributed the difficulty in defining influenza‐like illness to the dynamic change in symptoms throughout the course.^9^, ^10^ Influenza viruses tend to induce a more abrupt onset of symptoms^5^, ^11^ than respiratory syncytial virus or rhinoviruses.^12^ Whereas patients with influenza tend to have cough and fever simultaneously, patients with rhinovirus usually have cough after nasal symptoms.^13^ However, the detailed relationship between symptoms and influenza throughout the course of the disease has not been thoroughly investigated. Previous studies evaluating the association between the symptoms of influenza and the time course were either small virus challenge studies, retrospective patient medical record‐based studies, or studies with a significant amount of missing data.^9^, ^14^, ^15^

To explore the symptoms of influenza over time, we analyzed a dataset from an international multicenter prospective emergency department‐based influenza‐like illness cohort study.

## 2 METHOD

### 2.1 Study design and setting

Adult patients with fever and flu‐like symptoms were recruited in the emergency departments of a surveillance network (Johns Hopkins Centers of Excellence for Influenza Research and Surveillance) in the US and Taiwan between November 2015 and March 2020. This network contained four US hospitals and three hospitals in Taiwan, ranging from tertiary referral medical centers to regional hospitals (Table S3). This study was approved by the Institutional Review Board of Johns Hopkins University (IRB00135664, IRB00041233, IRB00141101, IRB00052743, and IRB00091667) and the Chang Gung Medical Foundation (201406930B0). We followed the Standard for Reporting Diagnostic Accuracy (STARD) for taking history and physical examination to report our manuscript.^16^

Adults aged greater than 18 years visited the emergency departments and met any of the inclusion criteria: (1) reported or measured fever and other flu‐like symptoms, (2) absence of any of the respiratory infection symptoms, or (3) positive laboratory test results for influenza from the current hospital visit. Flu‐like symptoms were defined as any of the three respiratory symptoms: cough, headache, and sore throat. Respiratory infection symptoms were defined as any of fever, cough, headache, sore throat, myalgia (unless due to trauma), rhinorrhea, nasal congestion, or shortness of breath. Eligible patients were interviewed by trained research coordinators using a pre‐specified and validated data collection form. Patients were excluded if they were unable to provide a written informed consent or had been previously enrolled in the study during the same influenza season. Patient demographics, comorbidities, history of influenza vaccination, pre‐defined symptoms, and days of illness were prospectively investigated by dedicated research coordinators. The first day of illness was defined as the day of symptom onset.

### 2.2 Laboratory analysis

Influenza virus infection was confirmed by portable polymerase chain reaction (PCR) testing using nasopharyngeal swabs immediately after the interview. The portable PCR‐based nucleic acid test was performed using the Xpert Flu Assay, a multiplex PCR platform (Cepheid). The overall sensitivity and specificity of this platform were both 98% and 100%.^17^ In addition, this platform could simultaneously subtype a positive influenza detection.

### 2.3 Statistical analysis

The differences in numerical means between the influenza‐positive and the influenza‐negative groups were evaluated using Wilcoxon's rank‐sum test or Student's *t*‐test and the categorical variables by the chi‐square test. In all investigations, two‐sided tests and *p*‐values of 0.05 were considered statistically significant. Odds ratios (OR) were used to measure the strength of the association between symptoms and influenza virus infection. We also measured the proportion of symptoms among influenza‐positive patients (sensitivity to detect influenza). To address the relationship between symptoms and influenza at different times, the days of illness were divided into quarters to evaluate the symptom dynamics at presentation. Univariate and multivariate logistic regression analyses were performed to adjust for confounding effects.

### 2.4 Subgroup and sensitivity analysis

To investigate the potential discrepancy, the analyses were repeated in the following subgroups: history of influenza vaccination, different countries (the US vs. Taiwan), and different dominant subtypes (H1N1 vs. H3N2). The dominant influenza subtypes were reported separately in the US and Taiwan during each influenza season. In the sensitivity analysis, the symptom dynamics among influenza‐positive patients were evaluated to determine the influence of different distributions of respiratory pathogens.

## 3 RESULT

### 3.1 Patient characteristics

**FIGURE 1:**
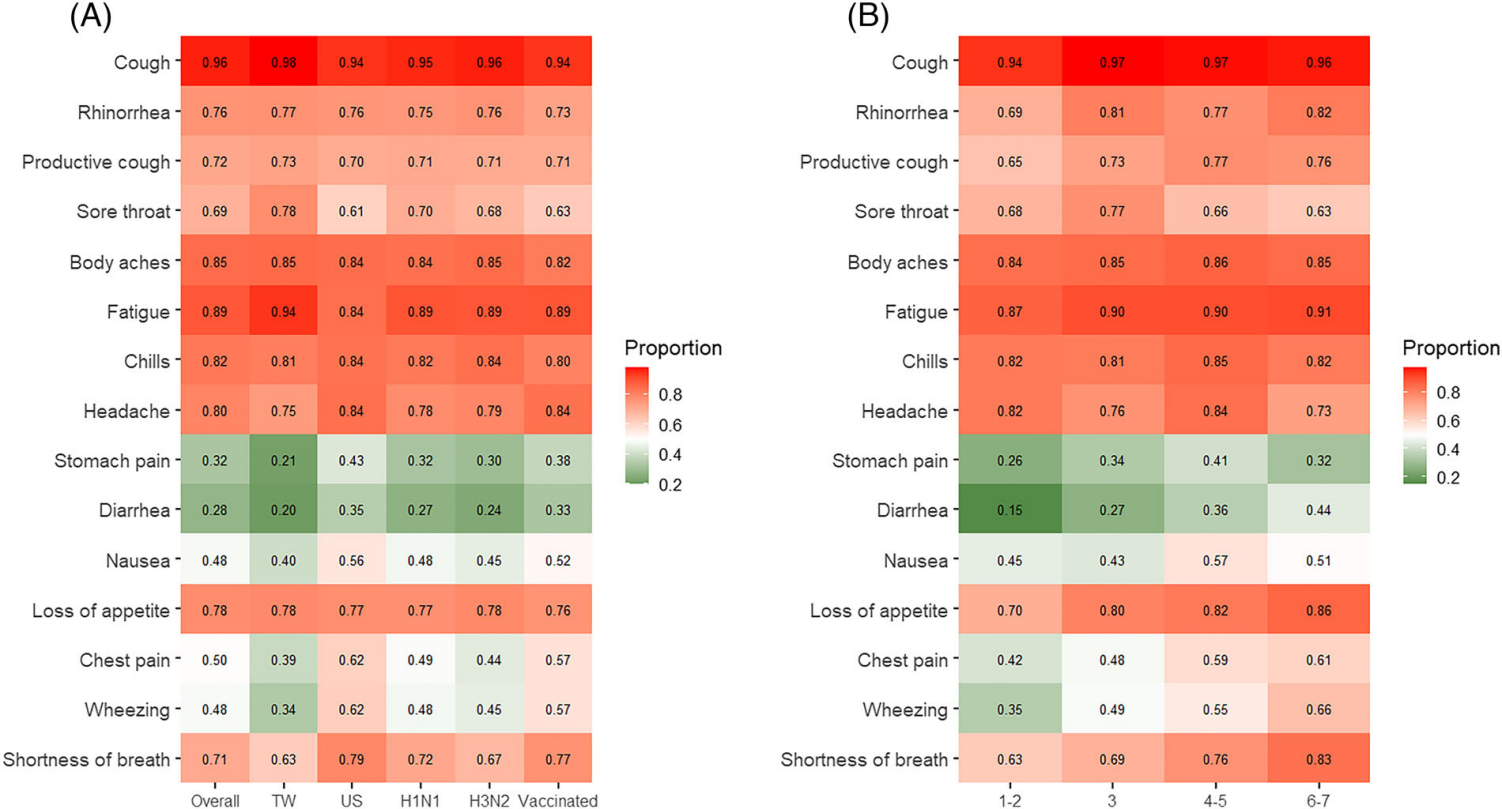
The proportion of symptoms in influenza‐positive patients in the overall group and different subgroups. (A) In general, cough was the most frequently reported symptom, followed by fatigue and body ache among patients tested positive for influenza virus infection. There was a higher portion of influenza patients that had lower respiratory symptoms among the US and the influenza‐vaccinated subgroup than the other subgroup. (B) Over 94% of the patients with influenza virus infection had cough throughout the week. About 85% of them had general symptoms and 70% of them had upper respiratory symptoms in the first half of the week. More than half of them had lower respiratory symptoms in the second half of the week. H1N1, H1N1‐dominant seasons; H3N2, H3N2‐dominant seasons; TW, Taiwan; US, the United States.

**FIGURE 2:**
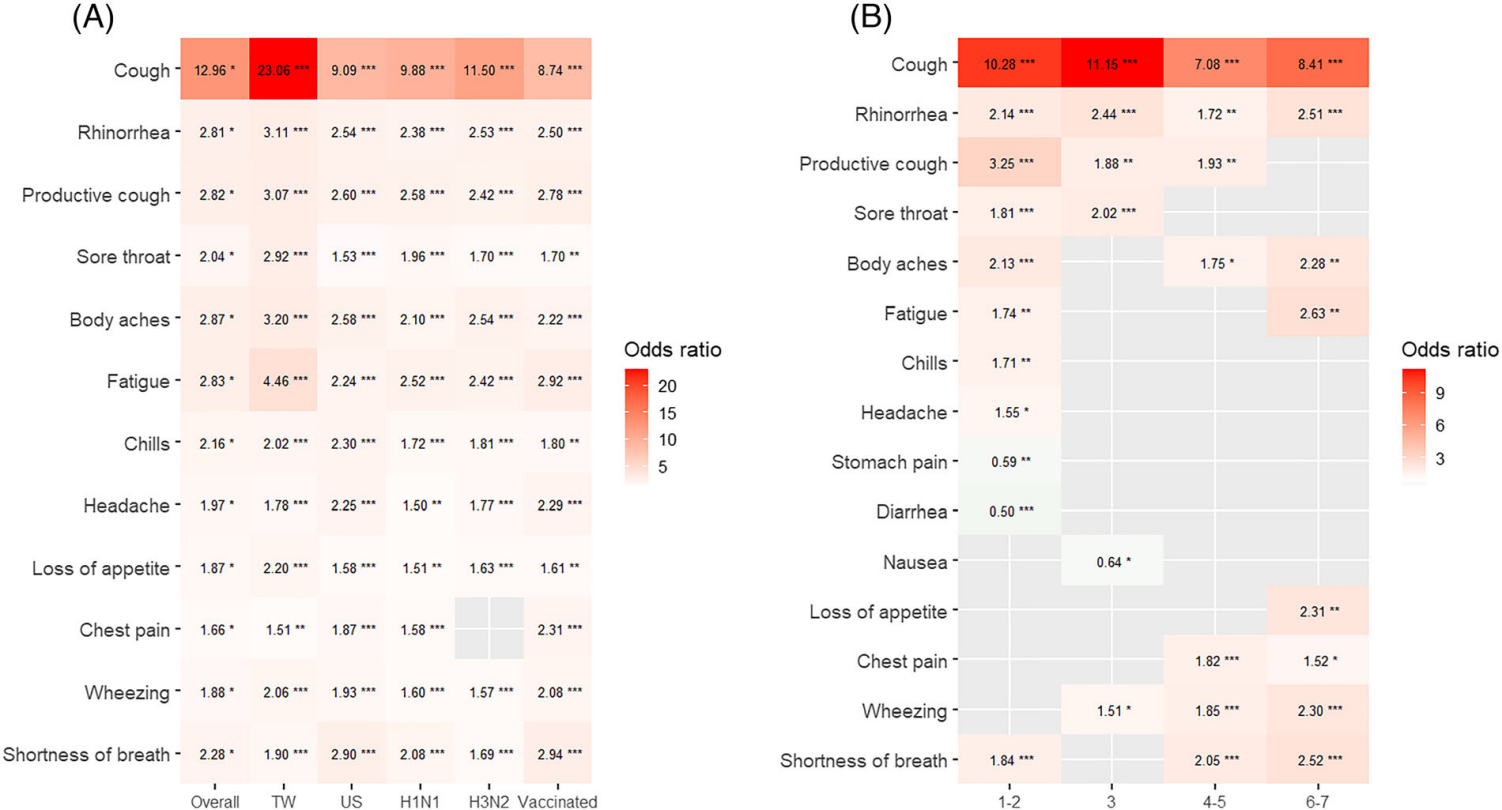
The performance of symptoms in predicting influenza virus infection in the overall group and different subgroups. (A) The odds ratios of symptom to predict influenza virus infection in the overall group and the different subgroups of patients. Cough was most predictive of influenza virus infection overall and in each subgroup. (B) The odds ratios of symptom to predict influenza virus infection in different days of illness. Cough was strongly predictive of influenza virus infection throughout the week. General symptoms (such as headache, chills, and fatigue) and some upper respiratory symptoms (such as sore throat and productive cough) were only predictive of influenza virus infection in the first half of the week. The lower respiratory symptoms (such as shortness of breath and wheezing) were predictive of influenza virus infection in the second half of the week. H1N1, H1N1‐dominant seasons; H3N2, H3N2‐dominant seasons; TW, Taiwan; US, the United States. Significance tests for the odds ratio were using logistic regression models: *p < 0.05, **p < 0.01, ***p < 0.001.

A total of 2471 patients were enrolled during five consecutive influenza seasons between 2015 and 2020, and half of the cases were enrolled in the US. In half of the patients aged greater than or equal to 39 years (interquartile range [IQR]: 29–53), 610 (24.7%) had chronic lung diseases, 324 (13.1%) had chronic liver diseases, 266 (10.8%) had cardiovascular diseases, and 172 (7%) had chronic kidney diseases. Among the enrolled patients, 994 (40.2%) tested positive for influenza virus infection, and 70% of them were recruited in the H1N1‐dominant seasons (Table 1, Table S4, and Table S5). Among patients who tested positive for influenza virus infection, cough was the most frequently reported symptom (sensitivity: 96.2%, 95% confidence interval [CI]: 94%–97.6%), followed by fatigue (88.9%, 95% CI: 85.7%–91.4%), and body ache (84.9%, 95% CI: 81.3%–87.8%, Figure 1A). Compared to patients who tested negative for influenza virus infection, patients with influenza virus infection were more likely to have cough (OR: 12.96, 95% CI: 9.28–18.10), fatigue (OR: 2.86, 95% CI: 2.27–3.6), and rhinorrhea (OR: 2.81, 95% CI: 2.35–3.36; Figure 2A).

**TABLE 1:** Characteristics of recruited patients

	Overall		Positive		Negative	
Median or *N* (IQR or %)	*N* = 2471		*N* = 994		*N* = 1477	
Demographics						
Male	1152	46.6	454	45.7	698	47.3
Age (year)***	39	(29–53)	41	(30–56)	37	(28–51)
Recruited in the US	1265	51.2	509	51.2	756	51.2
Recruited in H1N1‐dominant seasons**	1367	55.3	695	69.9	672	45.5
Vaccination*	772	31.2	282	28.4	490	33.2
Asian	1220	49.4	496	49.9	724	49
Black or African American	879	35.6	371	37.3	508	34.4
Caucasian	309	12.5	102	10.3	207	14
Comorbidities						
Chronic lung disease	610	24.7	266	26.8	344	23.3
End‐stage renal disease*	62	2.5	33	3.3	29	2
Liver cirrhosis*	32	1.3	20	2	12	0.8
Symptoms						
Days of illness**	3	(2–5)	3	(2–4)	4	(2–5)
Body temperature***	38.2	(37–39)	38.5	(37.8–39.1)	38	(36.6–38.8)
Fever**	2088	84.5	893	89.8	1195	80.9
Cough**	1911	77.3	954	96	957	64.8
Productive cough**	1412	57.1	713	71.7	699	47.3
Sore throat**	1462	59.2	688	69.2	774	52.4
Rhinorrhea**	1543	62.4	757	76.2	786	53.2
Headache**	1769	71.6	790	79.5	979	66.3
Body aches**	1818	73.6	843	84.8	975	66
Fatigue**	1976	80	884	88.9	1092	73.9
Chills**	1830	74.1	819	82.4	1011	68.4
Shortness breath**	1471	59.5	706	71	765	51.8
Wheezing**	973	39.4	481	48.4	492	33.3
Chest pain**	1064	43.1	502	50.5	562	38.1
Loss of appetite**	1739	70.4	774	77.9	965	65.3
Nausea	1162	47	480	48.3	682	46.2
Diarrhea	667	27	276	27.8	391	26.5
Stomach pain	826	33.4	323	32.5	503	34.1

*Note*: Demographic characteristics of adult patients' test for influenza virus infection from 2015 to 2020. Symptoms of patients with or without laboratory‐confirmed influenza virus infection.

Abbreviation: US, the United States.

* *p* < 0.05.

** *p* < 0.01.

*** *p* < 0.001.

### 3.2 Dynamics of predictive symptoms

The patients were further divided into four quartiles according to their days of illness during the first week of symptom onset (Day 1–2, 3, 4–5, and 6–7). More patients with influenza virus infection presented to our emergency departments during the first half of the week after symptom onset (1–3 days: 60.6%; Table S6). Most of the symptoms were predictive of influenza virus infection during the first 2 days of illness (OR: 1.55–10.28, Figure 2B). Among the 19 recorded symptoms, cough was the strongest predictor throughout the week (OR: 7.08–11.15, Figure 2B). Rhinorrhea was also predictive of influenza virus infection throughout the week (OR: 1.72–2.51, Figure 2B). However, general symptoms (headache, chills, and fatigue) and other upper respiratory symptoms (sore throat and productive cough) were only predictive of influenza virus infection during the first half of the week (1–3 days; OR: 1.55–3.25). During the second half of the week, lower respiratory symptoms were predictive of influenza virus infection, including shortness of breath, wheezing, and chest pain (4–7 days; OR: 2.52–1.52). We further adjusted for potential confounders, including age, race, end‐stage renal disease, and cirrhosis, and the adjusted OR of symptoms were not significantly different from the crude OR on different days of illness for predicting influenza virus infection (Figure S1).

### 3.3 Subgroup and sensitivity analysis

**FIGURE 3:**
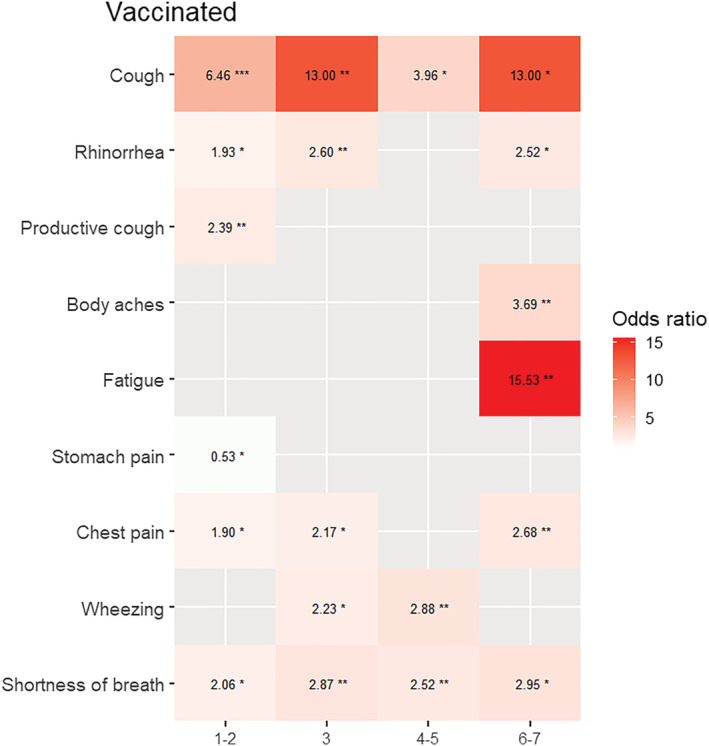
The odds ratio for symptoms of influenza virus infection with significance in vaccinated subgroup in the days of illness. Upper and lower respiratory symptoms were predictive of influenza throughout the week. General symptoms were not predictive of influenza in first half of the week. Significance tests for the odds ratio using logistic regression models: *p < 0.05, **p < 0.01, ***p < 0.001.

In the influenza‐vaccinated subgroup analysis, the vaccinated patients were older and had more comorbidities, including diabetes, end‐stage kidney disease, and asthma, than the unvaccinated patients (Table S7). In the influenza‐vaccinated subgroup, lower respiratory symptoms were predictive of influenza breakthrough infection throughout the week (shortness of breath, OR: 2.06–2.95, Figure 3), and general symptoms, such as headache and chills, were not predictive of influenza virus infection.

**FIGURE 4:**
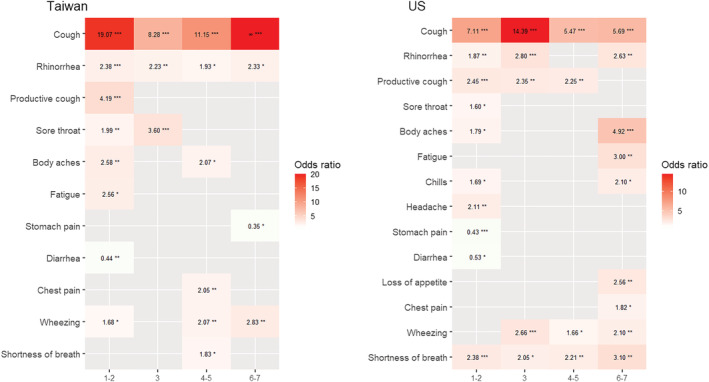
The odds ratio for symptoms of influenza virus infection with significance in the country‐specific subgroup analysis in the days of illness. The predictive symptoms in the first half of the week were almost similar in the two countries. Notably, the lower respiratory symptoms were predictive of influenza virus infection in the US throughout the week but not predictive of influenza virus infection in the early half of the week in Taiwan. US, the United States. Significance tests for the odds ratio using logistic regression models: *p < 0.05, **p < 0.01, ***p < 0.001. ^∞^Among 51 patients with influenza virus infection in Taiwan on the 6th and 7th day, all patients had the symptom of cough. Therefore, the estimated odds ratio was infinity.

**FIGURE 5:**
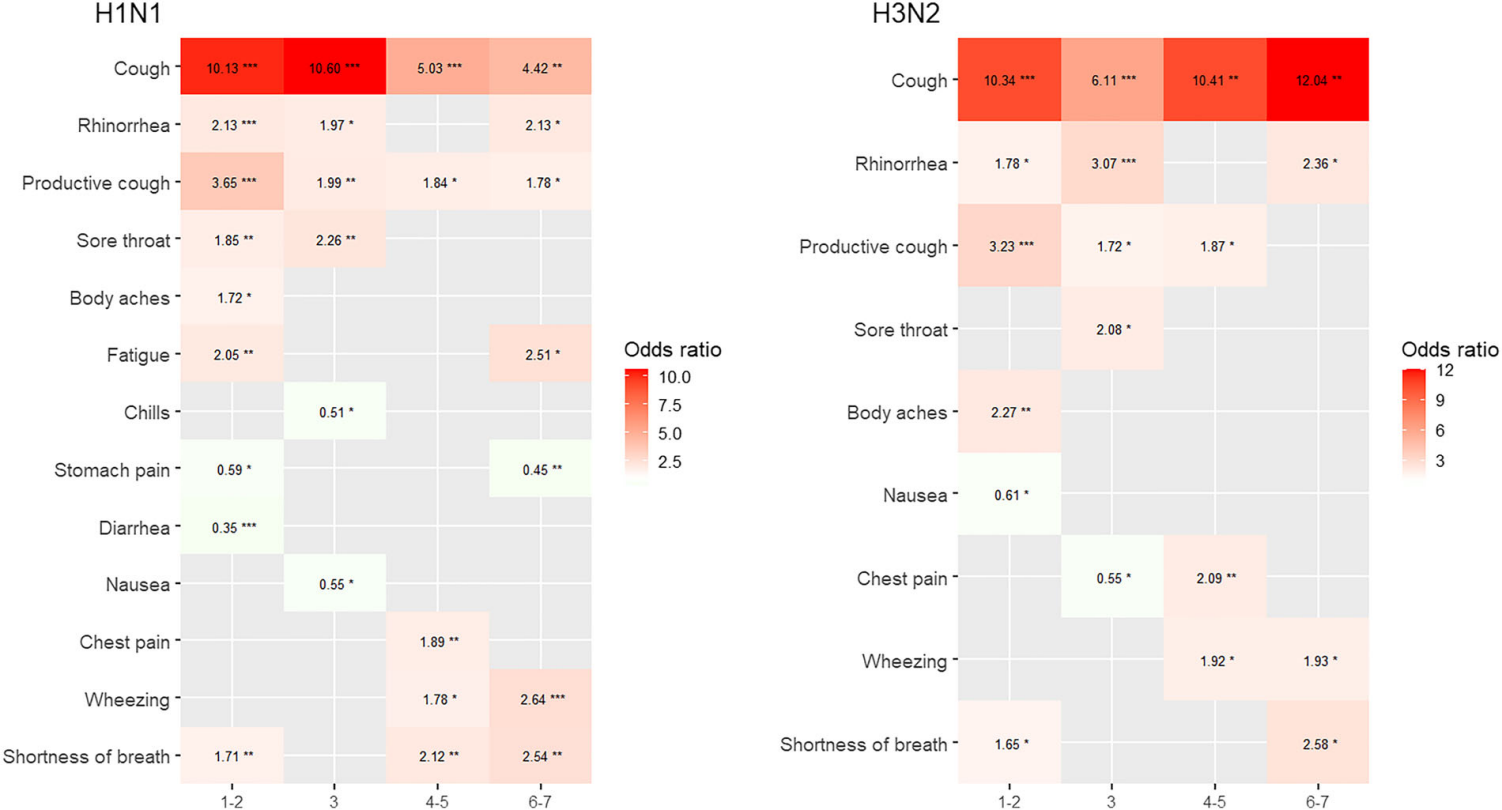
The odds ratio for symptoms of influenza virus infection with significance in the dominant subtype‐specific subgroup analysis in the days of illness. H1N1‐ and H3N2‐dominant season shared the symptoms predictive of influenza virus infection in common. Cough was strongly predictive of influenza virus infection throughout the week. Significance tests for the odds ratio using logistic regression models: *p < 0.05, **p < 0.01, ***p < 0.001.

In the country‐specific subgroup analysis, lower respiratory symptoms were predictive of influenza in the US throughout the week but not of influenza virus infection in the early half of the week in Taiwan (Figure 4 and Figure S2). In addition, the H3N2 subtypes were more commonly found in the Taiwanese subgroup (Table S8). In the subtype‐specific subgroup analyses, most of the symptoms predictive of influenza virus infection were similar among subtype H3N2 and H1N1 (Figure 5). However, after adjusting for confounders, lower respiratory symptoms were not predictive of influenza virus infection in H3N2‐dominant seasons after 5 days (Figure S3).

Among patients with influenza virus infection, symptom dynamics were found to be similar to the overall influenza‐like illness cohort. Similarly, patients with influenza virus infection tended to have cough throughout the week (94%–96%), sore throat in the first half of the week, and shortness of breath in the second half of the week (Figure 1B). Additionally, patients with influenza virus infection in the US tended to have more lower respiratory and less sore throat symptoms (Figure S4). Finally, breakthrough cases of influenza presented with increased symptoms of shortness of breath than the overall influenza‐like illness cohort throughout the week (Figure S5). However, the symptom dynamics of patients with influenza virus infection were similar in the H3N2‐dominant and H1N1‐dominant seasons (Figure S6).

## 4 DISCUSSION

In this prospective international multicenter emergency department‐based cohort study of patients with influenza‐like illness, the presentation of influenza was found to vary on different days of illness. Among patients with influenza virus infection, cough and rhinorrhea were more likely to appear consistently throughout the week, with cough being the most predictive symptom. Other upper respiratory symptoms, such as sore throat and productive cough, and general symptoms, such as body ache and fatigue, were more likely to appear in the first half of the week. Lower respiratory symptoms, such as shortness of breath and wheezing, appeared in the second half of the week. In our subgroup analysis, both subtypes of influenza A virus shared similar symptom dynamics with the overall cohort. However, lower respiratory symptoms were predictive of influenza virus infection in the vaccinated and US subgroups throughout the week. Nonetheless, lower respiratory symptoms were not predictive of influenza virus infection in the early half of the week in Taiwan.

From both clinical and public health perspectives, the early diagnosis of influenza virus infection is important, which could greatly impact patient management. Although rapid influenza diagnostic tools, such as point‐of‐care testing and reverse transcription‐polymerase chain reaction (RT‐PCR), are readily available, clinical gestalt for pre‐test probability is still required for sustainable health care and appropriate clinical pathways.^18^ To increase the pre‐test probability, many public health surveillance organizations have developed different definitions of influenza‐like illnesses in the past few decades.

Influenza‐like illness criteria have been developed to screen for potential influenza virus infection in many public health institutes, such as the CDC, ECDC, and WHO. Optimum case definitions should balance the sensitivity and specificity to capture more cases with an increasing number of false detections. The sensitivity of these influenza‐like illness criteria in many validation studies ranged from 31.5% to 96.1% (Table S2). In 2011, the WHO revised the influenza‐like illness defined in 1999 to overcome the low accuracy and different phenotypes of the novel H1N1 influenza A virus by omitting the sore throat and adding a time frame.^19^ In contrast, the ECDC version of influenza‐like illness had relatively high sensitivity but compromised specificity (96.1% vs. 6.6%, Table S2).^20^ However, the higher sensitivities of these different versions of influenza‐like illness were only found among patients who were enrolled earlier in their illness (Table S2).

Fever and cough were the most common symptoms in the different definitions of influenza‐like illness (Table S1). In our study, cough was not only found to be the most predictive symptom (OR: 12.96, 95% CI: 9.28–18.10) but also found to be a consistent predictor of influenza throughout the first week after symptom onset (OR: 7.08 to 11.5). Shah *et al* also reported that the combination of cough and fever was predictive of influenza virus infection in a retrospective electronic medical record‐based study consisting of patients who underwent influenza PCR testing (OR: 6.6).^21^ Furthermore, cough was also found to be the most predictive symptom to detect influenza virus infection in previous studies consisting of patients with any flu‐like symptoms (OR: 6.9 to 13).^4^, ^6^ The incidence of cough was higher among influenza patients than among other respiratory viruses, such as rhinovirus, respiratory syncytial virus, and coronavirus.^22^ A possible explanation may be the more predominant epithelial cell necrosis that damages airway integrity caused by influenza viruses, compared to other viruses such as rhinovirus and coronavirus.^23^

In our study, most of the upper respiratory and general symptoms reported in the first 2 days after symptom onset were more predictive of influenza virus infection than those reported in the latter days. Some researchers found that the symptom scores of patients with influenza virus and rhinovirus infection peak earlier than those of patients with respiratory syncytial virus infection after symptom onset in prospective studies (influenza and rhinoviruses: 2 days vs. respiratory syncytial virus: 5 days).^11^, ^12^ Woolpert *et al* also found that an abrupt onset of symptom within 3 days was more predictive of influenza virus infection (OR: 3.26).^24^ The results could be explained by the higher levels of cytokines, such as interferon‐α and interleukin‐6, that were found during the first 2 days of influenza virus infection in a virus challenge study.^11^

Nonetheless, considerable controversies exist between sore throat and influenza virus infections. Yang *et al* and Al‐Mahrezi *et al* pointed out that sore throat was associated with influenza virus infection (OR: 1.85 to 2.3),^25^, ^26^ whereas others found the opposite (OR: 0.41 to 0.72).^5^, ^27^ In 2011, the WHO removed sore throat from the influenza‐like illness criteria to increase the accuracy after the novel H1N1 influenza outbreak.^19^ Our data revealed that sore throat was more predictive of influenza virus infection in the first 3 days. In another prospective study, patients with influenza virus infection experienced sore throat in the earlier days (5.07 days vs. 6.42 days).^28^ We believe that the time factor could improve the accuracy of influenza virus infection prediction and could be used to modify influenza‐like illness criteria.

In our study, lower respiratory symptoms, such as shortness of breath, wheezing, and chest pain, were associated with influenza virus infection in the second half of the week. These symptoms are more likely to result from complications of influenza virus infection. The most common complication of influenza is pneumonia.^29^ The average interval between influenza virus infection and subsequent pneumonia was 6 days after infection, sooner than that for other respiratory viruses (14 days).^30^ Contrary to our results, shortness of breath was less likely to be found among patients with influenza virus infection who were recruited only within 3 days after the onset of fever in a prospective cross‐sectional study (OR: 0.4).^31^

In our subgroup analysis, some predictive symptoms differed between the US and Taiwan, after adjusting for potential confounders. These results could be partially explained by the different distributions of respiratory pathogens between the two countries. The most common pathogens of respiratory viral infections in the US are rhinovirus, influenza, and respiratory syncytial virus,^32^ whereas in Taiwan they are rhinovirus/enterovirus and adenovirus.^33^, ^34^ To avoid this spectrum bias, we examined the proportion (or sensitivity) of symptoms only among patients with influenza virus infection. Nevertheless, the sensitivity of symptoms still differed between the two countries after adjusting for possible confounders. A higher proportion of patients with influenza virus infection were found to have lower respiratory symptoms in the US than in Taiwan (Figure S4). Further studies with larger sample sizes are required to investigate the incidence of lower respiratory symptoms. We further performed dominant subtype‐specific subgroup analysis, in which no noticeable differences were found in predictive symptoms between H1N1 and H3N2 strain‐dominant seasons. In a systematic review of studies focusing on influenza subtypes, the symptoms of different influenza subtypes were also indistinguishable.^35^

Additionally, in our study, general symptoms were not predictive of influenza virus infection in the vaccinated subgroup. Similarly, patients who were vaccinated but still got infected with the influenza virus were significantly more likely to be afebrile with mild symptoms.^36^ These breakthrough cases of influenza virus infection were more likely to have shortness of breath than the overall study population throughout the week. We hypothesized that the lower respiratory symptoms of breakthrough cases could be attributed to their poor general health. Further research should be performed to clarify symptom dynamics among breakthrough cases of influenza.

Our study has several strengths. First, we conducted this multicenter international prospective cohort study with trained research coordinators using a pre‐specified data collection form to interview the patients in a consistent manner. Instead of retrospectively collecting symptoms documented in the medical records, our coordinators systematically inquired about every symptom that the patient might have. Second, we conducted this study across several consecutive influenza seasons, which enabled us to simultaneously compare different influenza subtypes. Third, we performed a multivariable analysis to avoid possible confounding factors in our study. Finally, to our knowledge, this is the largest study attempting to explore the time course of influenza symptoms.

Our study had several limitations. First, we modified the definition of influenza‐like illness in our eligibility criteria: fever and at least one of the three respiratory symptoms. Fever was deemed a powerful predictor of influenza virus infection in the past (OR: 3.47 to 4.03).^37^, ^38^ However, Smith *et al* noted that patients with afebrile influenza virus infection had fewer respiratory symptoms.^39^ To evaluate the potential bias of different influenza‐like illness definitions, we also recruited asymptomatic controls and a positive group. Further evaluation of symptoms throughout the course of influenza virus infection among afebrile patients remains limited. Second, we interviewed all patients only once during their visits to the emergency department, and the symptoms were accumulated upon enrollment, instead of obtaining a daily log of symptoms. Nonetheless, our results could be generalized to primary care settings, because primary physicians often could only evaluate accumulated symptoms upon arrival. Third, patients enrolled in the first half of the week were more likely to have influenza than those enrolled in the second half of the week. Theoretically, OR would overestimate the effect in populations with more events. Nevertheless, in the sensitivity analysis of patients with confirmed influenza virus infection, we still observed a dynamic trend of symptoms throughout the course. In addition, patients who recovered from respiratory virus infections in the second half of the week would not come to the emergency departments. However, patients who did not recover from influenza virus infection were more likely to have lower respiratory symptoms compared to other respiratory virus infections. Therefore, we recommend that the frontline health care providers still should consider influenza virus infection among patients with lower respiratory symptoms during the second half of the week. Finally, our surveillance network included multiple sites in Taiwan and the US. The corresponding pathogens in non‐influenza‐infected patients with flu‐like symptoms could still influence the diagnostic OR of symptoms. Although we checked the sensitivity of the symptoms among influenza‐infected patients, discrepant results still existed in these two countries. Similarly, we cannot completely attribute the different symptom distributions to different influenza subtypes. Nonetheless, the time course still seems to be an important factor in evaluating symptoms throughout the course of the infection in different countries and seasons.

In conclusion, the time course is an essential factor to be considered when attempting to predict influenza virus infection by symptoms. Cough and rhinorrhea were associated with influenza in the first week. Other upper respiratory and general symptoms were associated with influenza in the first half of the week, whereas lower respiratory symptoms were associated with influenza in the second half of the week. Understanding the time course of influenza symptoms would be helpful for clinicians to treat patients with influenza‐like illnesses.

### 4.1 Protocol

The study protocol is available upon request. The brief description can be found at https://www.niaidceirs.org/resources/cohort-studies/.

## CONFLICT OF INTEREST

The authors have no conflict of interest to declare.

## ETHICS STATEMENT

This study was approved by the Institutional Review Board of Johns Hopkins University (IRB00135664, IRB00041233, IRB00141101, IRB00052743, and IRB00091667) and the Chang Gung Medical Foundation (201406930B0).

## AUTHOR CONTRIBUTIONS

**Jin‐Hua Li:** Writing‐original draft. **Chin‐Chieh Wu:** Formal analysis; writing‐review and editing. **Yi‐Ju Tseng:** Conceptualization; data curation; funding acquisition; writing‐review and editing. **Shih‐Tsung Han:** Supervision; writing‐review and editing. **Andrew Pekosz:** Data curation; supervision; writing‐review and editing. **Richard E Rothman:** Data curation; supervision; writing‐review and editing. **Kuan‐Fu Chen:** Conceptualization; data curation; funding acquisition; project administration; writing‐review and editing.

## PATIENT CONSENT STATEMENT

Patients who could not provide written informed consent, who were currently incarcerated, or who were previously enrolled in the study during the same influenza season were excluded. After informed consent, patient demographic information, comorbidities, history of exposure to a confirmed influenza virus infection patient in the past 5 days, travel, date of emergency department visit, and clinical symptoms were collected by research coordinators as the feature candidates. The coordinators also obtained nasopharyngeal swab for the influenza PCR test.

## PERMISSION TO REPRODUCE MATERIAL FROM OTHER SOURCES

No data used in this study have been included as part of another manuscript (whether published, submitted, or in preparation).

### PEER REVIEW

The peer review history for this article is available at https://publons.com/publon/10.1111/irv.13081.

## Supporting information


**Figure S1.** the odds ratio for symptoms of influenza virus infection with significance with or without adjustment in overall patients in the days of illness

**Figure S2.** the odds ratio for symptoms of influenza virus infection with significance with or without adjustment in the country‐specific subgroup analysis in the days of illness

**Figure S3.** the odds ratio for symptoms of influenza virus infection with significance with or without adjustment in the dominant subtype‐specific subgroup analysis in the days of illness

**Figure S4.** The proportion of symptoms in influenza‐positive patients in the country‐specific subgroup analysis in the days of illness

**Figure S5.** The proportion of symptoms in breakthrough cases of influenza viruses

**Figure S6.** The proportion of symptoms in influenza‐positive patients in the dominant subtype‐specific subgroup analysis in the days of illness

**Table S1.** The definition of influenza‐like illness in different public health surveillance organizations
**Table S2.** The performance of influenza‐like illness
**Table S3.** Information about JHCEIRS (Johns Hopkins Centers of Excellence for Influenza Research and Surveillance) network hospitals in the US and Taiwan.
**Table S4.** Patients recruited in different arms in the cohort
**Table S5.** Dominant influenza subtype in different countries and seasons
**Table S6.**the number of influenza‐positive and ‐negative patients in the quartile of the first week of symptom onset
**Table S7.** Characteristics of recruited patients grouped by vaccination status
**Table S8.** Association between countries and strain‐dominant seasons

## Data Availability

The datasets generated during and/or analyzed during the current study are available from the corresponding author on reasonable request.

## References

[1] World Health O . Global influenza strategy 2019–2030. World Health Organization 0919, 2021. Updated 2019. https://apps.who.int/iris/handle/10665/311184

[2] Cate TR . Clinical manifestations and consequences of influenza. Am J Med. 1987;82(6, Supplement 1):15‐19. doi:10.1016/0002-9343(87)90555-9 3591813

[3] Friedman MJ , Attia MW . Clinical predictors of influenza in children. Arch Pediatr Adolesc Med. 2004;158(4):391‐394. doi:10.1001/archpedi.158.4.391 15066881

[4] Yang J‐H , Huang P‐Y , Shie S‐S , et al. Predictive symptoms and signs of laboratory‐confirmed influenza: a prospective surveillance study of two metropolitan areas in Taiwan. Medicine. 2015;94(44):e1952. doi:10.1097/md.0000000000001952 26554802 PMC4915903

[5] Monto AS , Gravenstein S , Elliott M , Colopy M , Schweinle J . Clinical signs and symptoms predicting influenza infection. Arch Intern Med. 2000;160(21):3243‐3247. doi:10.1001/archinte.160.21.3243 11088084

[6] Lam P‐P , Coleman BL , Green K , et al. Predictors of influenza among older adults in the emergency department. BMC Infect Dis. 2016;16(1):615. doi:10.1186/s12879-016-1966-4 27793117 PMC5084347

[7] Nichol KL , D'Heilly S , Ehlinger EP . Influenza vaccination among college and university students: impact on influenzalike illness, health care use, and impaired school performance. Arch Pediatr Adolesc Med. 2008;162(12):1113‐1118. doi:10.1001/archpedi.162.12.1113 19047537

[8] Call SA , Vollenweider MA , Hornung CA , Simel DL , McKinney WP . Does this patient have influenza? Jama. 2005;293(8):987‐997. doi:10.1001/jama.293.8.987 15728170

[9] Han A , Poon J‐L , Powers JH , Leidy NK , Yu R , Memoli MJ . Using the influenza patient‐reported outcome (FLU‐PRO) diary to evaluate symptoms of influenza viral infection in a healthy human challenge model. BMC Infect Dis. 2018;18(1):353. doi:10.1186/s12879-018-3220-8 30055573 PMC6064178

[10] Otera H , Yamamoto G , Matsubara K , et al. Clinical study of the time course of clinical symptoms of pandemic (H1N1) 2009 influenza observed in young adults during an initial epidemic in Kobe, Japan. Intern Med. 2011;50(11):1163‐1167. doi:10.2169/internalmedicine.50.4723 21628930

[11] Canini L , Carrat F . Population modeling of influenza a/H1N1 virus kinetics and symptom dynamics. J Virol. 2011;85(6):2764‐2770. doi:10.1128/JVI.01318-10 21191031 PMC3067928

[12] Tyrrell DA , Cohen S , Schlarb JE . Signs and symptoms in common colds. Epidemiol Infect. 1993;111(1):143‐156. doi:10.1017/s0950268800056764 8394240 PMC2271186

[13] Bennett JE , Dolin R , Blaser MJ . Mandell, Douglas, and Bennett's principles and practice of infectious diseases E‐book. Elsevier Health Sci. 2019;2143‐2168:2262‐2268.

[14] Choi SH , Lee MS , Park KH , Kim T , Kwak YG , Chung JW . Late diagnosis of influenza in adult patients during a seasonal outbreak. Korean J Intern Med. 2018;33(2):391‐396. doi:10.3904/kjim.2016.226 29117669 PMC5840597

[15] Murray EL , Khagayi S , Ope M , et al. What are the most sensitive and specific sign and symptom combinations for influenza in patients hospitalized with acute respiratory illness? Results from western Kenya, January 2007–July 2010. Epidemiol Infect. 2013;141(1):212‐222. doi:10.1017/S095026881200043X 22417876 PMC9152063

[16] Simel DL , Rennie D , Bossuyt PM . The STARD statement for reporting diagnostic accuracy studies: application to the history and physical examination. J Gen Intern Med. 2008;23(6):768‐774. doi:10.1007/s11606-008-0583-3 18347878 PMC2517891

[17] Novak‐Weekley SM , Marlowe EM , Poulter M , et al. Evaluation of the Cepheid Xpert flu assay for rapid identification and differentiation of influenza a, influenza a 2009 H1N1, and influenza B viruses. J Clin Microbiol. 2012;50(5):1704‐1710. doi:10.1128/JCM.06520-11 22378908 PMC3347140

[18] Lee BY , McGlone SM , Bailey RR , et al. To test or to treat? An analysis of influenza testing and antiviral treatment strategies using economic computer modeling. PLoS ONE. 2010;5(6):e11284. doi:10.1371/journal.pone.0011284 20585642 PMC2890406

[19] Fitzner J , Qasmieh S , Mounts AW , et al. Revision of clinical case definitions: influenza‐like illness and severe acute respiratory infection. Bull World Health Organ. 2018;96(2):122‐128. doi:10.2471/BLT.17.194514 29403115 PMC5791775

[20] Casalegno J‐S , Eibach D , Valette M , et al. Performance of influenza case definitions for influenza community surveillance: based on the French influenza surveillance network GROG, 2009–2014. Euro Surveill. 2017;22(14):30504. doi:10.2807/1560-7917.ES.2017.22.14.30504 28422004 PMC5388124

[21] Shah SC , Rumoro DP , Hallock MM , et al. Clinical predictors for laboratory‐confirmed influenza infections: exploring case definitions for influenza‐like illness. Infect Control Hosp Epidemiol. 2015;36(3):241‐248. doi:10.1017/ice.2014.64 25695163

[22] Lin L , Yang Z‐F , Zhan Y‐Q , et al. The duration of cough in patients with H1N1 influenza. Clin Respir J. 2017;11(6):733‐738. doi:10.1111/crj.12409 26519198 PMC7162306

[23] Winther B , Gwaltney JM , Hendley JO . Respiratory virus infection of monolayer cultures of human nasal epithelial cells. Am Rev Respir Dis. 1990;141(4 Pt 1):839‐845. doi:10.1164/ajrccm/141.4_Pt_1.839 2158258

[24] Woolpert T , Brodine S , Lemus H , Waalen J , Blair P , Faix D . Determination of clinical and demographic predictors of laboratory‐confirmed influenza with subtype analysis. BMC Infect Dis. 2012;12(1):129. doi:10.1186/1471-2334-12-129 22676850 PMC3407722

[25] Yang TU , Cheong HJ , Song JY , et al. Age‐ and influenza activity‐stratified case definitions of influenza‐like illness: experience from hospital‐based influenza surveillance in South Korea. PLoS ONE. 2014;9(1):e84873. doi:10.1371/journal.pone.0084873 24475034 PMC3901651

[26] Al‐Mahrezi A , Samir N , Al‐Zakwani I , Al‐Muharmi Z , Balkhair A , Al‐Shafaee M . Clinical characteristics of influenza a H1N1 versus other influenza‐like illnesses amongst outpatients attending a university health center in Oman. Int J Infect Dis. 2012;16(7):e504‐e507. doi:10.1016/j.ijid.2012.02.015 22521779

[27] Ohmit SE , Monto AS . Symptomatic predictors of influenza virus positivity in children during the influenza season. Clin Infect Dis. 2006;43(5):564‐568. doi:10.1086/506352 16886147

[28] Mullins J , Cook R , Rinaldo C , Yablonsky E , Hess R , Piazza P . Influenza‐like illness among university students: symptom severity and duration due to influenza virus infection compared to other etiologies. J am Coll Health. 2011;59(4):246‐251. doi:10.1080/07448481.2010.502197 21308583 PMC4944816

[29] Yokomichi H , Mochizuki M , Lee JJ , Kojima R , Yokoyama T , Yamagata Z . Incidence of hospitalisation for severe complications of influenza virus infection in Japanese patients between 2012 and 2016: a cross‐sectional study using routinely collected administrative data. BMJ Open. 2019;9(1):e024687. doi:10.1136/bmjopen-2018-024687 PMC6340484 30782739

[30] Prasso JE , Deng JC . Postviral complications: bacterial pneumonia. Clin Chest Med. 2017;38(1):127‐138. doi:10.1016/j.ccm.2016.11.006 28159155 PMC5324726

[31] Anderson KB , Simasathien S , Watanaveeradej V , et al. Clinical and laboratory predictors of influenza infection among individuals with influenza‐like illness presenting to an urban Thai hospital over a five‐year period. PLoS ONE. 2018;13(3):e0193050. doi:10.1371/journal.pone.0193050 29513698 PMC5841736

[32] Mandelia Y , Procop GW , Richter SS , Worley S , Liu W , Esper F . Dynamics and predisposition of respiratory viral co‐infections in children and adults. Clin Microbiol Infect. 2021;27(4):631.e1‐631.e6. doi:10.1016/j.cmi.2020.05.042 32540470

[33] Shih H‐I , Wang H‐C , Su I‐J , et al. Viral respiratory tract infections in adult patients attending outpatient and emergency departments, Taiwan, 2012–2013: a PCR/electrospray ionization mass spectrometry study. Medicine. 2015;94(38):e1545‐e1545. doi:10.1097/MD.0000000000001545 26402811 PMC4635751

[34] Chiu S‐C , Lin Y‐C , Wang H‐C , et al. Surveillance of upper respiratory infections using a new multiplex PCR assay compared to conventional methods during the influenza season in Taiwan. Int J Infect Dis. 2017;61:97‐102. doi:10.1016/j.ijid.2017.06.011 28625839 PMC7110889

[35] Caini S , Kroneman M , Wiegers T , El Guerche‐Séblain C , Paget J . Clinical characteristics and severity of influenza infections by virus type, subtype, and lineage: a systematic literature review. Influenza Other Respi Viruses. 2018;12(6):780‐792. doi:10.1111/irv.12575 PMC6185883 29858537

[36] Deiss RG , Arnold JC , Chen W‐J , et al. Vaccine‐associated reduction in symptom severity among patients with influenza a/H3N2 disease. Vaccine. 2015;33(51):7160‐7167. doi:10.1016/j.vaccine.2015.11.004 26562321 PMC4684491

[37] Domínguez À , Soldevila N , Torner N , et al. Usefulness of clinical definitions of influenza for public health surveillance purposes. Viruses. 2020;12(1):95. doi:10.3390/v12010095 31947696 PMC7019582

[38] Falsey AR , Baran A , Walsh EE . Should clinical case definitions of influenza in hospitalized older adults include fever? Influenza Other Respi Viruses. 2015;9(S1):23‐29. doi:10.1111/irv.12316 PMC4549099 26256292

[39] Smith BJ , Price DJ , Johnson D , et al. Influenza with and without fever: clinical predictors and impact on outcomes in patients requiring hospitalization. Open Forum Infect Dis. 2020;7(7). doi:10.1093/ofid/ofaa268 PMC7580166 33123614

