# Supplementary figures and images for: Applying symptom dynamics to accurately predict influenza virus infection: An international multicenter influenza‐like illness surveillance study
Source: Influenza Other Respir Viruses. 2022 Dec 8;17(1):e13081. doi: 10.1111/irv.13081 (PMC9835452; doi:10.1111/irv.13081)

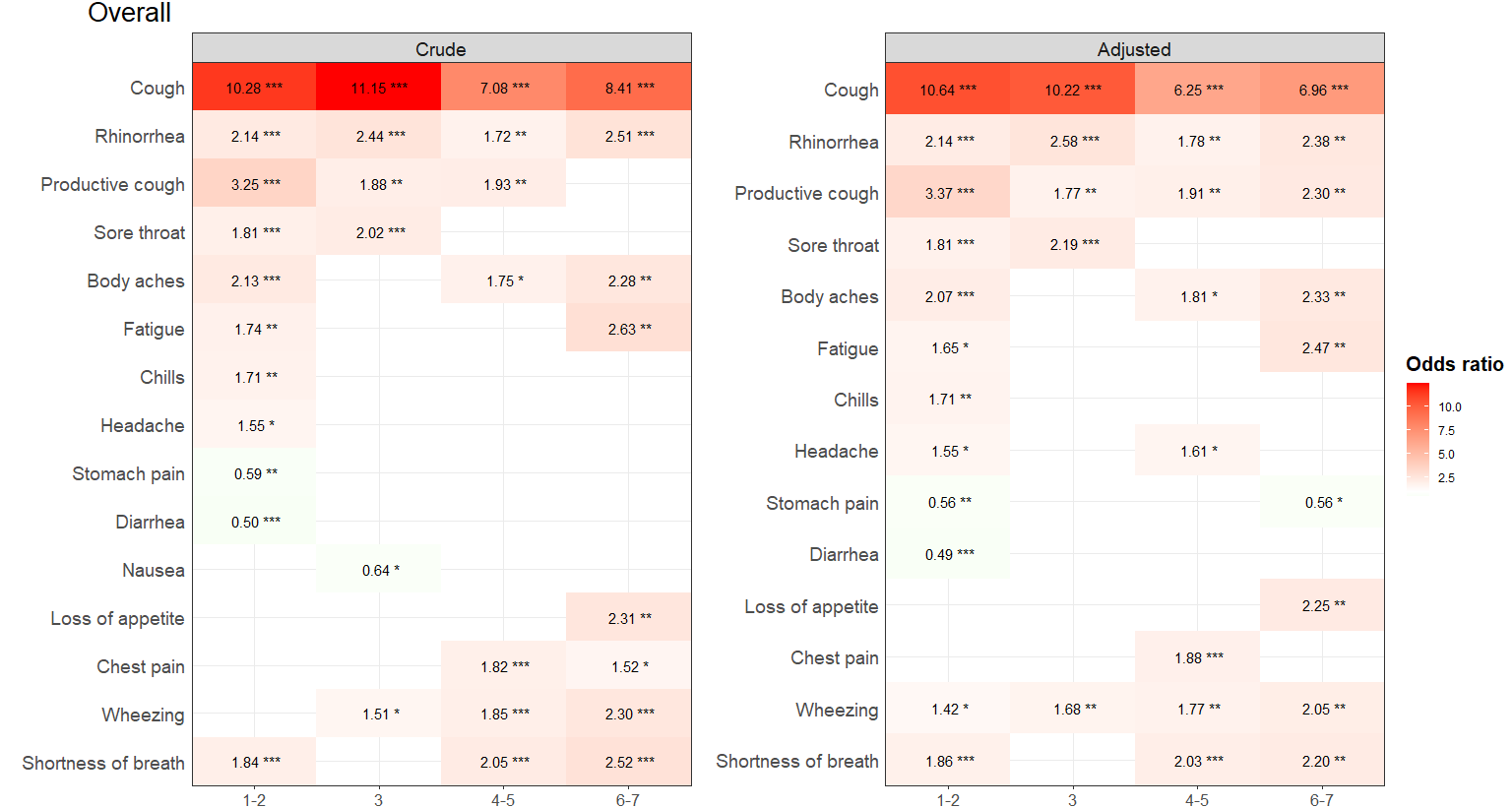

Supplement: Supplementary file 1 — Figure S1. the odds ratio for symptoms of influenza virus infection with significance with or without adjustment in overall patients in the days of illness [file IRV-17-e13081-s002.tiff]

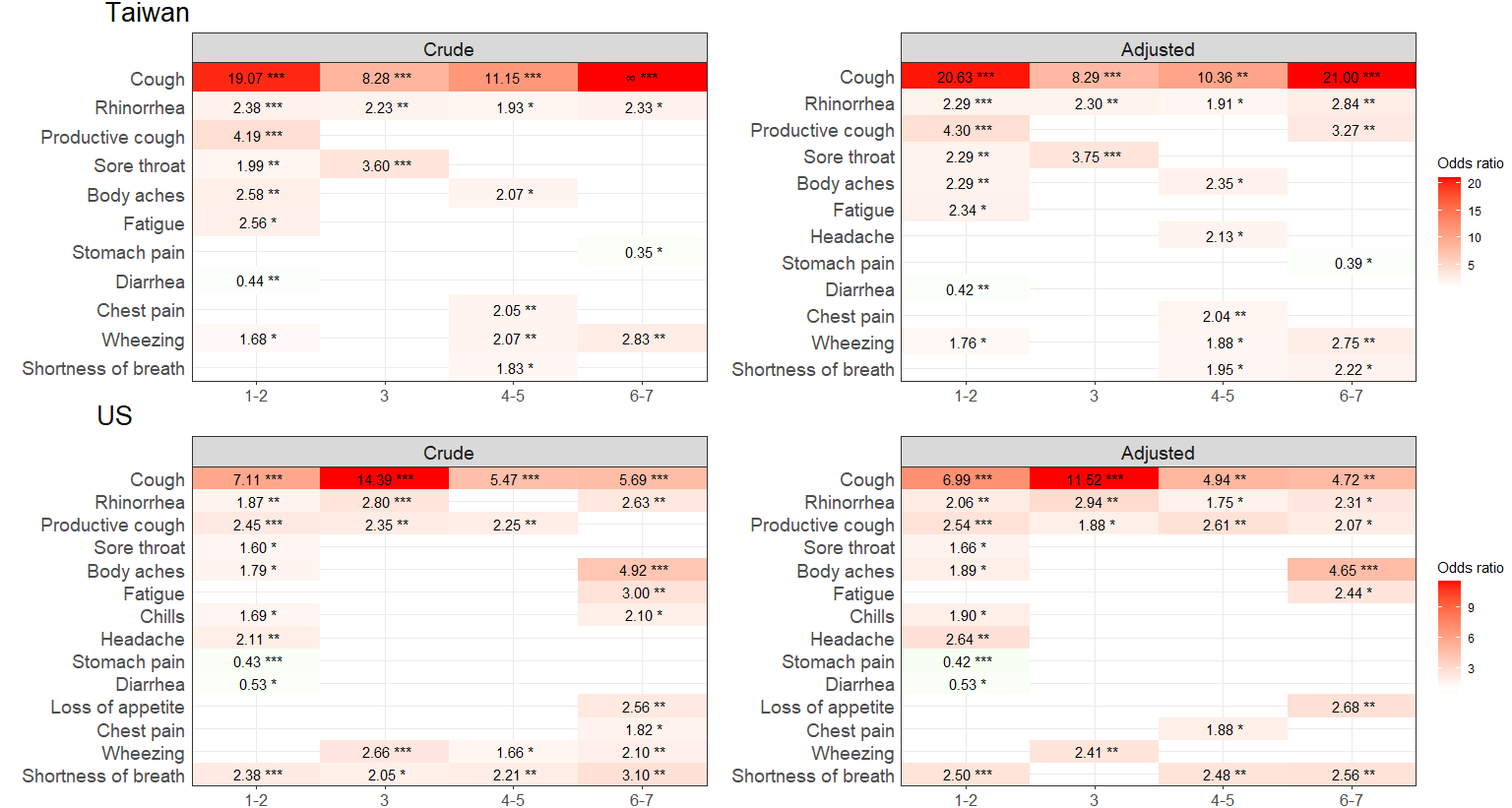

Supplement: Supplementary file 2 — Figure S2. the odds ratio for symptoms of influenza virus infection with significance with or without adjustment in the country‐specific subgroup analysis in the days of illness [file IRV-17-e13081-s005.tiff]

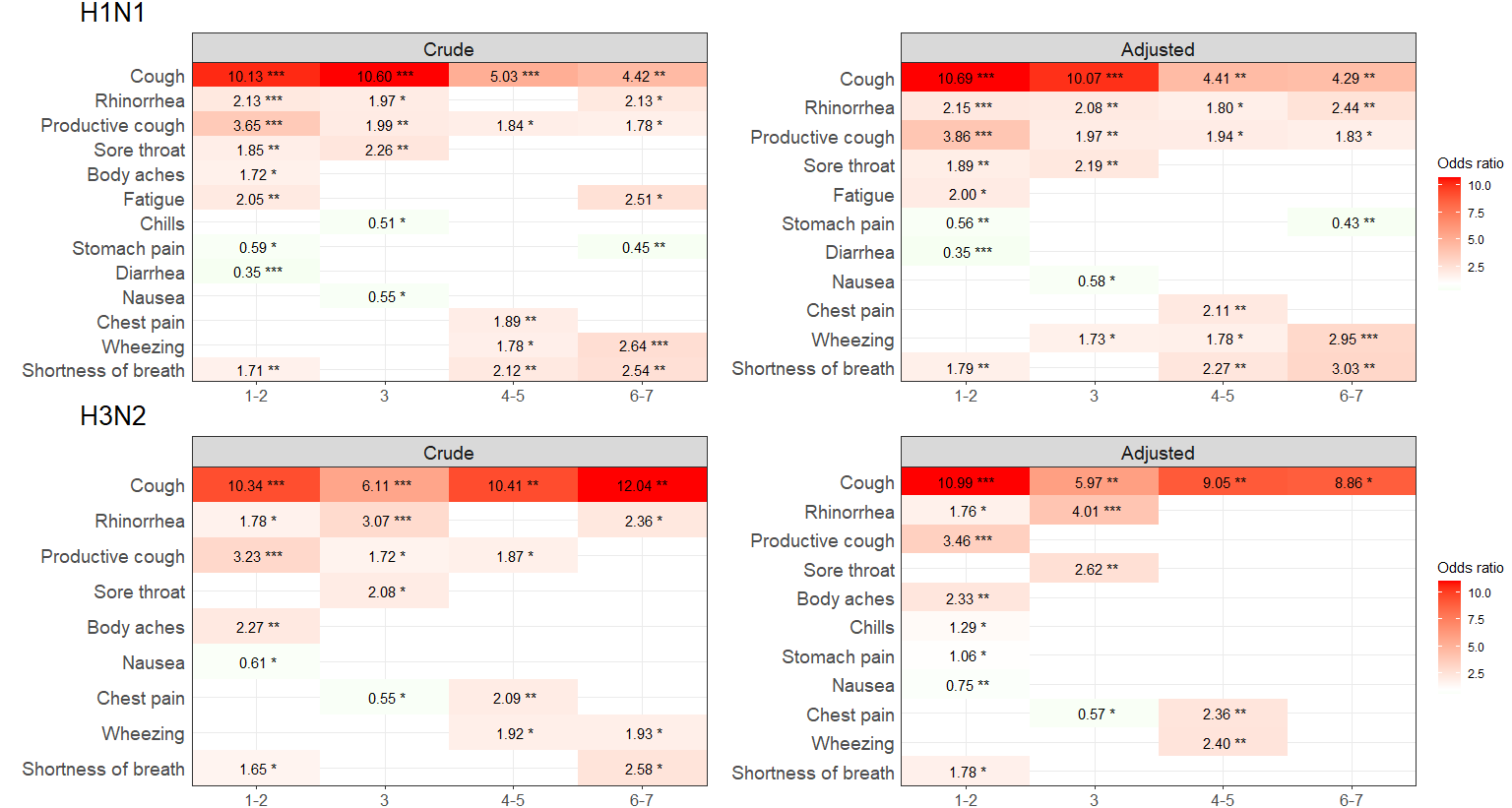

Supplement: Supplementary file 3 — Figure S3. the odds ratio for symptoms of influenza virus infection with significance with or without adjustment in the dominant subtype‐specific subgroup analysis in the days of illness [file IRV-17-e13081-s004.tiff]

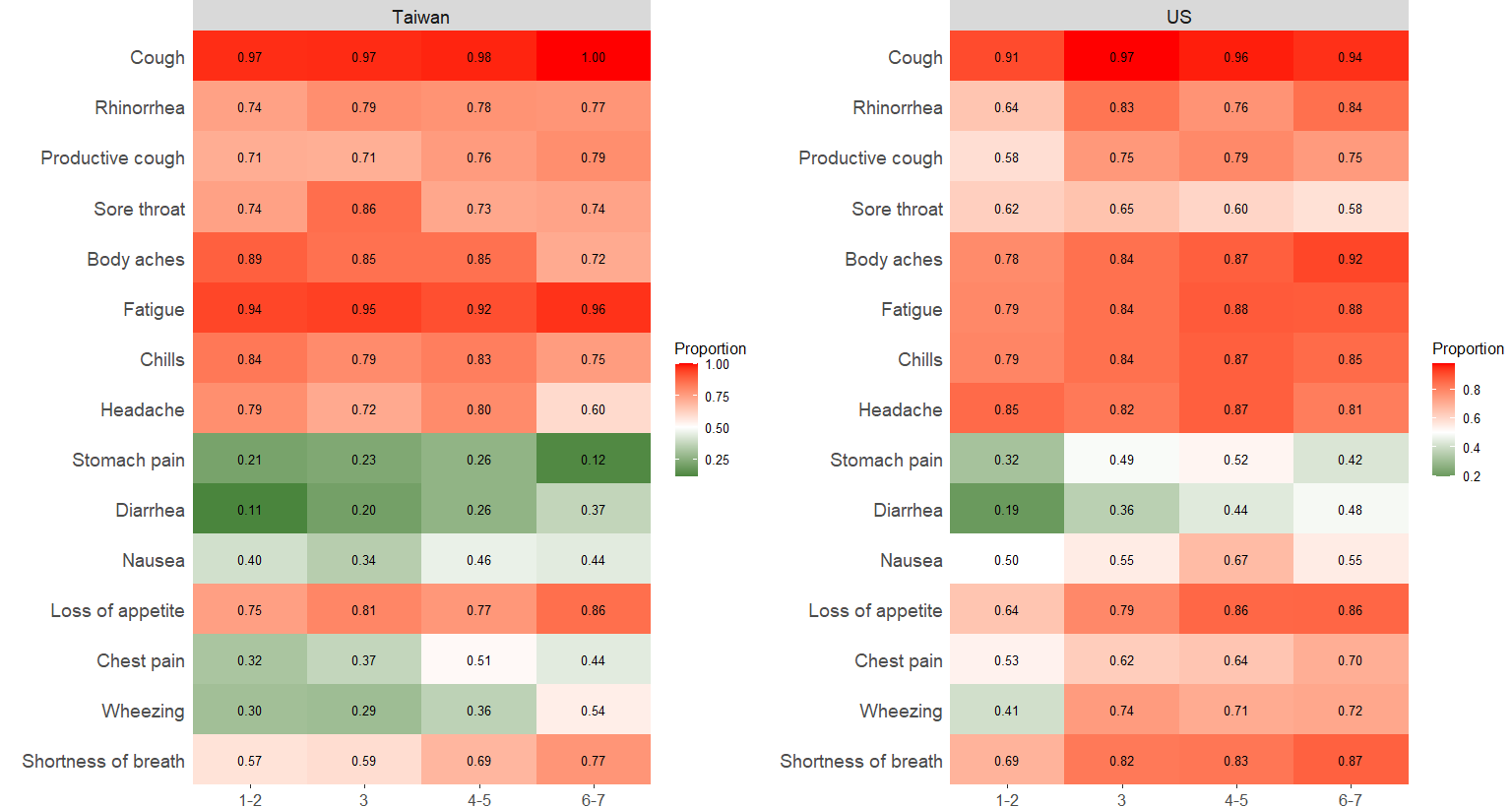

Supplement: Supplementary file 4 — Figure S4. The proportion of symptoms in influenza‐positive patients in the country‐specific subgroup analysis in the days of illness [file IRV-17-e13081-s001.tiff]

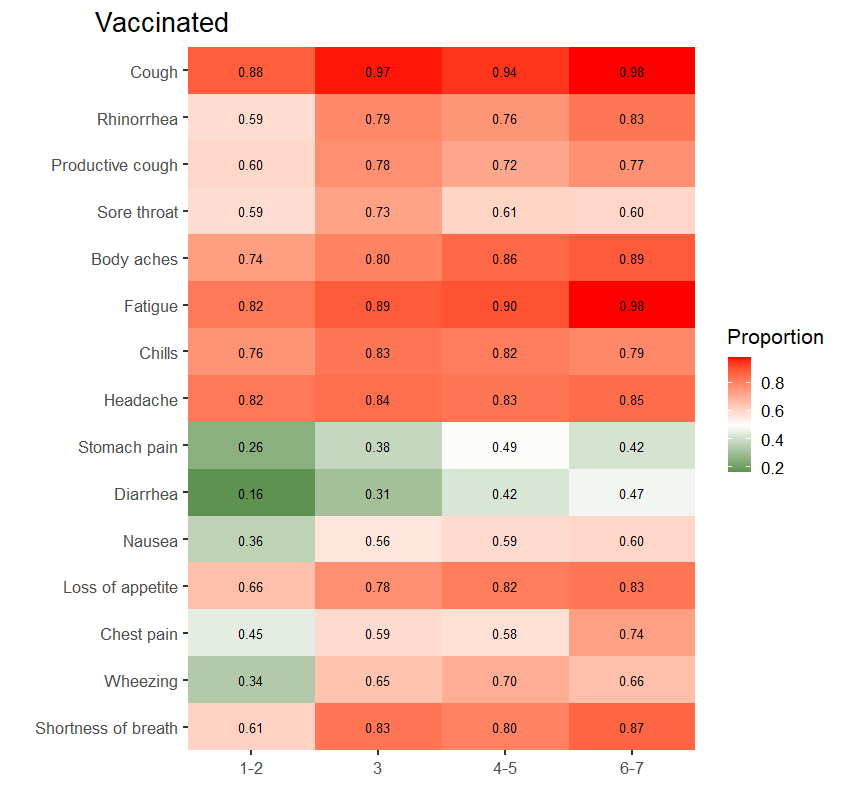

Supplement: Supplementary file 5 — Figure S5. The proportion of symptoms in breakthrough cases of influenza viruses [file IRV-17-e13081-s006.tiff]

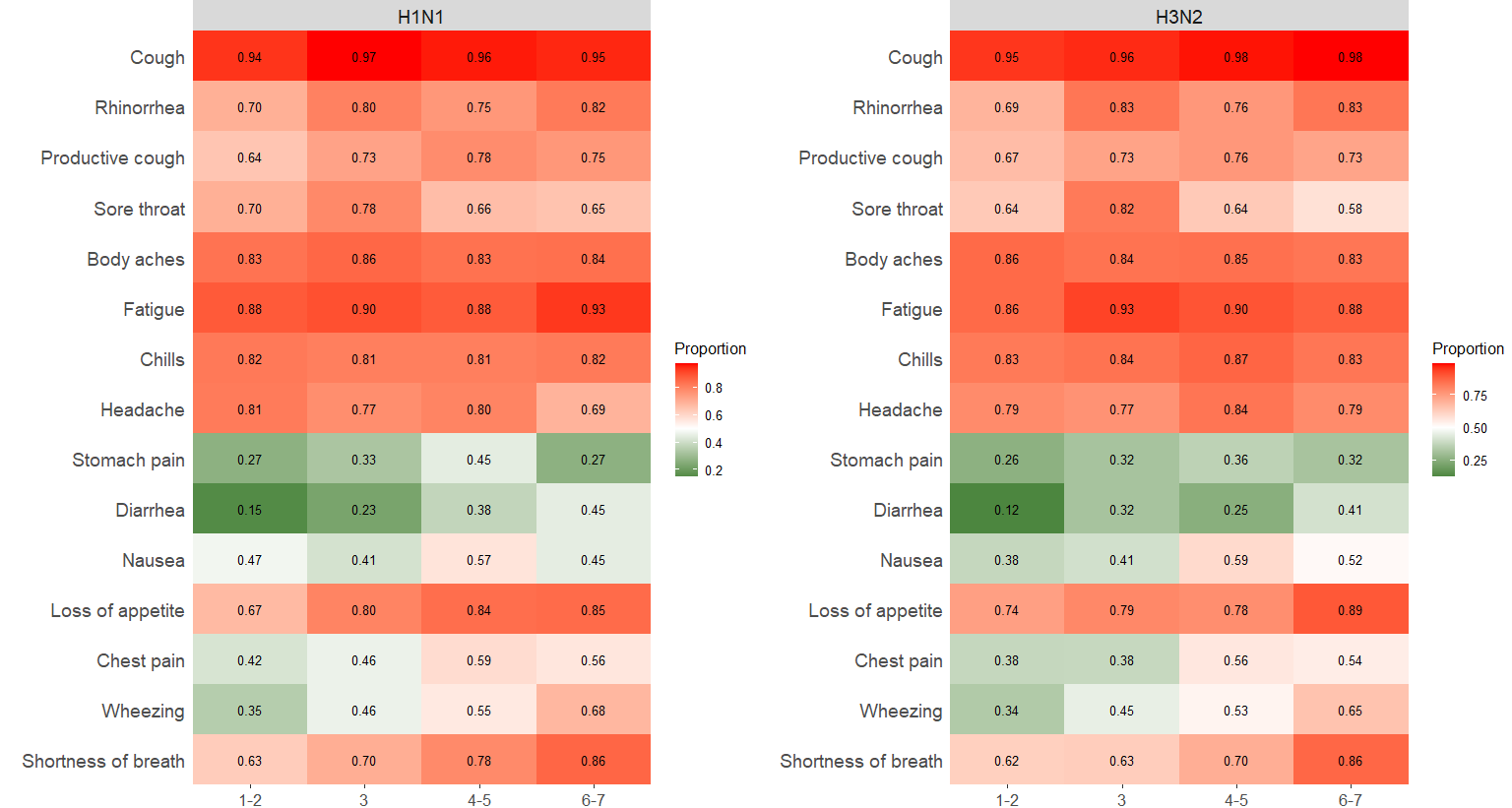

Supplement: Supplementary file 6 — Figure S6. The proportion of symptoms in influenza‐positive patients in the dominant subtype‐specific subgroup analysis in the days of illness [file IRV-17-e13081-s003.tiff]
