# Supplementary material for: Applying symptom dynamics to accurately predict influenza virus infection: An international multicenter influenza‐like illness surveillance study
Source: Influenza Other Respir Viruses. 2022 Dec 8;17(1):e13081. doi: 10.1111/irv.13081 (PMC9835452; doi:10.1111/irv.13081)
Supplement: Supplementary file 7 — Table S1. The definition of influenza‐like illness in different public health surveillance organizations Table S2. The performance of influenza‐like illness Table S3. Information about JHCEIRS (Johns Hopkins Centers of Excellence for Influenza Research and Surveillance) network hospitals in the US and Taiwan. Table S4. Patients recruited in different arms in the cohort Table S5. Dominant influenza subtype in different countries and seasons Table S6.the number of influenza‐positive and ‐negative patients in the quartile of the first week of symptom onset Table S7. Characteristics of recruited patients grouped by vaccination status Table S8. Association between countries and strain‐dominant seasons [file IRV-17-e13081-s007.docx]

Supplementary Table 1. The definition of influenza-like illness in different public health surveillance organizations

| Organization | ILI definition |
| --- | --- |
| ECDC ^1^ | Sudden onset of symptoms AND  at least one of the following four systemic symptoms:   - Fever or feverishness - Malaise - Headache - Myalgia AND   At least one of the following three respiratory symptoms:   - Cough, - Sore throat - Shortness of breath |
| CDC ^2^ | fever (temperature of 100°F [37.8°C] or greater) AND  cough AND/OR sore throat |
| WHO in 1999 ^3^ | a sudden onset of fever, a temperature >38°C AND  cough OR  sore throat AND  in the absence of another diagnosis |
| WHO in 2011 ^4^ | An acute respiratory illness with a measured temperature of ≥ 38 ̊C AND cough, AND  with onset within the past 7 days. |

Supplementary table 2. The performance of influenza-like illness

| ILI def/study | sensitivity | specificity | Age | The time of symptom onset |
| --- | --- | --- | --- | --- |
| ECDC | | | | |
| Casalegno 2017 ^5^ | 96.1% | 6.6% | half of patients aged 18 years or older | 48 hr |
| Domínguez 2020 ^6^ | 58% | 50% | half of patients aged 15 years or older | ^NA^ |
| CDC | | | | |
| Shah 2015 ^7^ | 90% | 43% | 86% of patients aged 50 years or younger | ^NA^ |
| Casalegno 2017  ^5^ | 95.7% | 7.3% | half of patients aged 18 years or older | 48 hr |
| DeMarcus 2018  ^8^ | 78.9% | 42.4% | ^NA^ | 72hr |
| Yang 2015  ^9^ | 87% | 40% | adult patients | 72hr |
| WHO in 1999 | | | | |
| Kasper 2010  ^10^ | 68% | 53% | adult patients | 72hr |
| Murray 2013  ^11^ | 31.5% | 74.9% | 33% of patients aged 5 years or older | 90% in 7 days |
| WHO in 2011 | | | | |
| Casalegno 2017^5^ | 89.8% | 21.4% | half of patients aged 18 years or older | 48 hr |
| Domínguez 2020 ^6^ | 82% | 33% | half of patients aged 15 years or older | Sudden onset^†^ |
| Yang 2015 ^9^ | 85% | 63% | adult patients | 72hr |

NA: not available

^†^ the enrollment citeria included sudden onset of symptom based on ECDC influenza-like illness definition

Reference:

1. EU case definitions. Official Journal of the European Union. https://eur-lex.europa.eu/legal-content/EN/TXT/PDF/?uri=CELEX:32018D0945&from=EN#page=24. Published 2018. Accessed August 18, 2021.

2. Centers for Disease Control and Prevention. Overview of influenza surveillance in the United States. https://www.cdc.gov/flu/weekly/overview.htm. Updated 20201206. Accessed.

3. World Health O. WHO recommended surveillance standards. In. 2nd ed ed. Geneva: World Health Organization; 1999.

4. Organization WH. WHO global technical consultation: global standards and tools for influenza surveillance. World Health Organization, . http://apps.who.int/iris/bitstream/handle/10665/70724/WHO_HSE_GIP_2011.1_eng.pdf;jsessionid=E3FE0C23DF082494AD8FFA56E6633E1E?sequence=1. Published 2011. Accessed August 18, 2021, 2021.

5. Casalegno JS, Eibach D, Valette M, et al. Performance of influenza case definitions for influenza community surveillance: based on the French influenza surveillance network GROG, 2009-2014. *Euro Surveill.* 2017;22(14):30504.

6. Domínguez À, Soldevila N, Torner N, et al. Usefulness of Clinical Definitions of Influenza for Public Health Surveillance Purposes. *Viruses.* 2020;12(1):95.

7. Shah SC, Rumoro DP, Hallock MM, et al. Clinical predictors for laboratory-confirmed influenza infections: exploring case definitions for influenza-like illness. *Infect Control Hosp Epidemiol.* 2015;36(3):241-248.

8. DeMarcus LS, Soderlund LV, Voss JD. Assessment of 12 influenza-like illness case definitions using Department of Defense Global, Laboratory-based Influenza Surveillance Program data, 2011-2014. *Msmr.* 2018;25(1):10-15.

9. Yang J-H, Huang P-Y, Shie S-S, et al. Predictive Symptoms and Signs of Laboratory-confirmed Influenza: A Prospective Surveillance Study of Two Metropolitan Areas in Taiwan. *Medicine.* 2015;94(44):e1952.

10. Kasper MR, Wierzba TF, Sovann L, Blair PJ, Putnam SD. Evaluation of an influenza-like illness case definition in the diagnosis of influenza among patients with acute febrile illness in Cambodia. *BMC infectious diseases.* 2010;10:320-320.

11. Murray EL, Khagayi S, Ope M, et al. What are the most sensitive and specific sign and symptom combinations for influenza in patients hospitalized with acute respiratory illness? Results from western Kenya, January 2007–July 2010. *Epidemiol Infect.* 2013;141(1):212-222.

Supplementary table 3.

Information about JHCEIRS (Johns Hopkins Centers of Excellence for Influenza Research and Surveillance) network hospitals in the US and Taiwan

|  | bed count | status |
| --- | --- | --- |
| US | | |
| Johns Hopkins Hospital | 1177 beds with 100,000 annual ED visits | a tertiary referral medical center located in urban area |
| Johns Hopkins Bayview Medical Center | 448 beds with 60,000 annual ED visits | a regional hospital located in suburb area of a metropolitan city |
| Taiwan | | |
| Linkou Chang Gung Memorial Hospital | 3406 beds with 160,000 annual ED visits | a tertiary referral medical center located in urban area |
| Keelung Chang Gung Memorial Hospital | 1089 beds with 68,000 annual ED visits | a regional hospital located in suburb area of a metropolitan city |
| Taipei Chang Gung Memorial Hospital | 252 beds with 17,000 annual ED visits | a community hospital located in capital city of Taiwan |

Supplementary Table 4 Patients recruited in different arms in the cohort

|  | Influenza Positive (N=994) | Influenza Negative (N=1473) |
| --- | --- | --- |
| Prospective Symptomatic | 407 (40.9%) | 1199 (81.4%) |
| Prospective Asymptomatic | 1 (0.1%) | 274 (18.6%) |
| Positive group | 586 (59%) | 0 (0.0%) |

Supplementary Table 5. Dominant Influenza subtype in different countries and seasons

| Influenza season | Dominant Subtype | |
| --- | --- | --- |
|  | Taiwan | US |
| 2015-2016 | H1N1 & B | H1N1 |
| 2016-2017 | H3N2 | H3N2 |
| 2017-2018 | B | H3N2 |
| 2018-2019 | H3N2 & H1N1 | H1N1 & H3N2 |
| 2019-2020 | H1N1 | B & H1N1 |

Supplementary Table 6. the number of influenza-positive and -negative patients in the quartile of the first week of symptom onset

| Influenza | Day of illness | | | |  |
| --- | --- | --- | --- | --- | --- |
|  | 1-2 | 3 | 4-5 | 6-7 | Total |
| Positive | 334 (48.13%) | 268 (49.08%) | 229 (38.42%) | 163 (25.75%) | 994 (40.26%) |
| Negative | 360 (51.87%) | 278 (50.92%) | 367 (61.58%) | 470 (74.25%) | 1475 (59.74%) |
| Total | 694 | 546 | 596 | 633 | 2469 |
| number | Pearson $X^{2}$(3) = 91.78, p-value < 0.001 | | | | NA=2 |

Supplementary Table 7. Characteristics of recruited patients grouped by vaccination status

|  |  | vaccinated | | Non-vaccinated | |
| --- | --- | --- | --- | --- | --- |
| Median or N (IQR or %) | | N=772 |  | N=1699 |  |
| Demographics | |  |  |  |  |
| male | | 337 | (43.6) | 819 | (48.1) |
| age | | 45 | (31-58) | 37 | (28-50) |
| Influenza positive | | 282 | 36.5 | 712 | 41.9 |
| Recruited in the US | | 180 | 23.3 | 1025 | 60.4 |
| Recruited in H1N1 dominant seasons | | 390 | 50.5 | 977 | 57.5 |
| comorbidities | |  |  |  |  |
| asthma | | 206 | 20.7 | 259 | 17.5 |
| End Stage Renal Disease | | 33 | 3.3 | 29 | 2 |
| cirrhosis | | 20 | 2 | 12 | 0.8 |
| Diabetes | | 141 | 14.2 | 166 | 11.2 |

Supplementary Table 8. Association between countries and strain-dominant seasons

|  |  | |  |
| --- | --- | --- | --- |
| Country | H1N1 | H3N2 | Total number |
| CGMH | 612  (50.75%) | 594  (49.26%) | 1206 (48.81%) |
| JHHS | 716  (56.60%) | 549  (43.40%) | 1265 (51.19%) |
| Total number | 1328  (53.74%) | 1143  (46.26%) | 2471 |
|  | Pearson $X^{2}$(1)=8.5123, p-value=0.004 | |  |
